# Soft tissue deformation for surgical simulation: a position-based dynamics approach

**DOI:** 10.1007/s11548-016-1373-8

**Published:** 2016-03-19

**Authors:** Mafalda Camara, Erik Mayer, Ara Darzi, Philip Pratt

**Affiliations:** Department of Surgery and Cancer, Imperial College London, London, UK

**Keywords:** Biomechanical modelling, Soft tissue deformation, Position-based dynamics, Robot-assisted partial nephrectomy, Ultrasound simulation

## Abstract

**Purpose:**

To assist the rehearsal and planning of robot-assisted partial nephrectomy, a real-time simulation platform is presented that allows surgeons to visualise and interact with rapidly constructed patient-specific biomechanical models of the anatomical regions of interest. Coupled to a framework for volumetric deformation, the platform furthermore simulates intracorporeal 2D ultrasound image acquisition, using preoperative imaging as the data source. This not only facilitates the planning of optimal transducer trajectories and viewpoints, but can also act as a validation context for manually operated freehand 3D acquisitions and reconstructions.

**Methods:**

The simulation platform was implemented within the GPU-accelerated NVIDIA FleX position-based dynamics framework. In order to validate the model and determine material properties and other simulation parameter values, a porcine kidney with embedded fiducial beads was CT-scanned and segmented. Acquisitions for the rest position and three different levels of probe-induced deformation were collected. Optimal values of the cluster stiffness coefficients were determined for a range of different particle radii, where the objective function comprised the mean distance error between real and simulated fiducial positions over the sequence of deformations.

**Results:**

The mean fiducial error at each deformation stage was found to be compatible with the level of ultrasound probe calibration error typically observed in clinical practice. Furthermore, the simulation exhibited unconditional stability on account of its use of clustered shape-matching constraints.

**Conclusions:**

A novel position-based dynamics implementation of soft tissue deformation has been shown to facilitate several desirable simulation characteristics: real-time performance, unconditional stability, rapid model construction enabling patient-specific behaviour and accuracy with respect to reference CT images.

**Electronic supplementary material:**

The online version of this article (doi:10.1007/s11548-016-1373-8) contains supplementary material, which is available to authorized users.

## Purpose

Robot-assisted partial nephrectomy (RAPN) is a surgical procedure that potentially benefits from organ modelling and patient-specific simulation due to the inherent anatomical complexity, specifically, the highly variable vascular and tumour anatomy. The rehearsal and planning of such a procedure should ultimately lead to an improved performance in the operating room, a decrease in operating times and intraoperative rate of errors, and to increase the ability of surgeons to complete the procedure [1].

Isotani et al. [2] developed a simulation approach for patient-specific planning of RAPN, but the system remains incapable of real-time navigation, tissue interaction or deformation by the user. Makiyama et al. [3] developed a patient-specific simulator for preoperative planning and training of renal surgery. However, this system has not focused on modelling the relevant aspects of a partial nephrectomy, as the system simulates radical nephrectomy. Figueroa et al. [4] developed a biomechanical model of the kidney to predict the estimated tumour displacement with respect to the kidney surface in the presence of an external load. In RAPN the surgeon loses tactile feedback through palpation and is often forced to resort to intraoperative ultrasound in order to discriminate between healthy and malignant tissue. This need for ultrasound scanning has encouraged the development of simulation-based environments [5–7], with some focused on replicating ultrasound images in the presence of deformation. The platform presented in this paper aims to provide a framework for volumetric deformation, allowing the visualisation and interaction with a biomechanical model of soft tissue. A patient-specific biomechanical model of the patient’s anatomical regions of interest is implemented through a position-based dynamics (PBD) approach. Furthermore, this allows the simulation of patient-specific intracorporeal 2D ultrasound image acquisition, using preoperative imaging as the input data.

The approaches used for the simulation of deformable bodies have been mainly focused on physically based frameworks. Traditional examples range from finite element methods, mass–spring systems, meshless methods to particle systems. A review of such approaches can be found in [8]. These physically based approaches model deformable bodies through the manipulation of internal and external forces. Forces are transformed via the mass of constituent parts into accelerations, using Newton’s second law of motion. The elements that comprise an object move to a certain position at each time step, determined by an integration scheme that computes the current position from the derived accelerations.

A PBD approach, unlike the aforementioned methods, models objects through the manipulation of position displacements to solve geometrical constraints. In contrast to force-based methods that achieve equilibrium configurations through the integration of accelerations, this geometrically based approach directly projects positions as a solution to a quasi-static problem. In a PBD approach, an object is composed of multiple particles and by manipulating the constraint functions of the system, one can model different types of material properties and behaviours. A shape-matching technique is an example of such a constraint function that can be used to model rigid bodies, providing visually plausible behaviours. This algorithm is efficient, stable and straightforward to implement. However, it can only accommodate small deformations, and to account for larger movement, i.e. to model soft tissue, a supplementary cluster-based deformation can be integrated [9]. The advantages of using this type of implementation are robustness, simplicity, visual accuracy, real-time performance, efficiency and controllability [10]. This geometrically motivated and mesh-free concept has been used to model animations in computer graphics due to their appealing performance and visual capabilities in real time, assuring a stable simulation and maintaining low computational times. The PBD approach has already been applied in the medical field. Kubiak et al. [11] developed a real-time surgical thread simulation, for an interactive and robust simulation of knot tying. The work of Wang et al. [12] couples a mass–spring model with a shape-matching technique to achieve a fast and stable simulation in virtual reality systems, focusing on the deformation of a heart model. As the scope of this research is related to the development of a robotic surgery simulation platform, where all feedback is visual, it is reasonable to prioritise visual fidelity over having precise deformation accuracy. However, the recent work developed by Bender et al. [13] demonstrates that through modelling and optimising the simulation parameters or coupling the simulation with a continuum-based formulation, complex physical phenomena can be accurately exhibited.

The implementation developed in this paper aims to present a framework that allows for a plausible and realistic deformation of soft tissue, thereby making possible the implementation of ultrasound simulation, using preoperative imaging as the source of anatomical data. The use of the PBD approach with a clustered shape-matching constraint implementation is novel in the field of soft tissue surgical simulation environments.

## Methods

### Experimental set-up

**Fig. 1:**
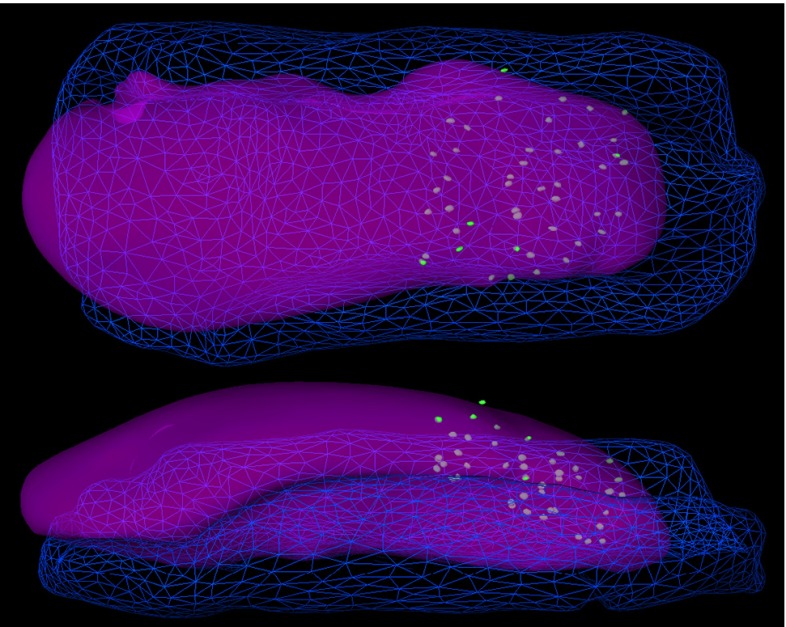
Volumetric distribution of the fiducials embedded within the kidney

CT images were acquired with a GE Innova 4100 scanner. The hardware specifications used for performing the simulation were a HP Z820 machine and an NVIDIA K5000 GPU processor with 1536 cores. CT scan acquisitions from a porcine kidney under different levels of deformation were carried out to be used as the ground truth for the biomechanical behaviour of soft tissue. Fiducial glass beads with a diameter of 1.5 mm was embedded within the porcine kidney. A total of 45 beads was distributed in multiple nylon threads and introduced with a needle throughout the kidney. Knots were regularly distributed on the threads to place the beads inside but allowing them to move freely relative to each other. The volumetric distribution of the beads in the simulation environment is illustrated in Fig. 1.

**Fig. 2:**
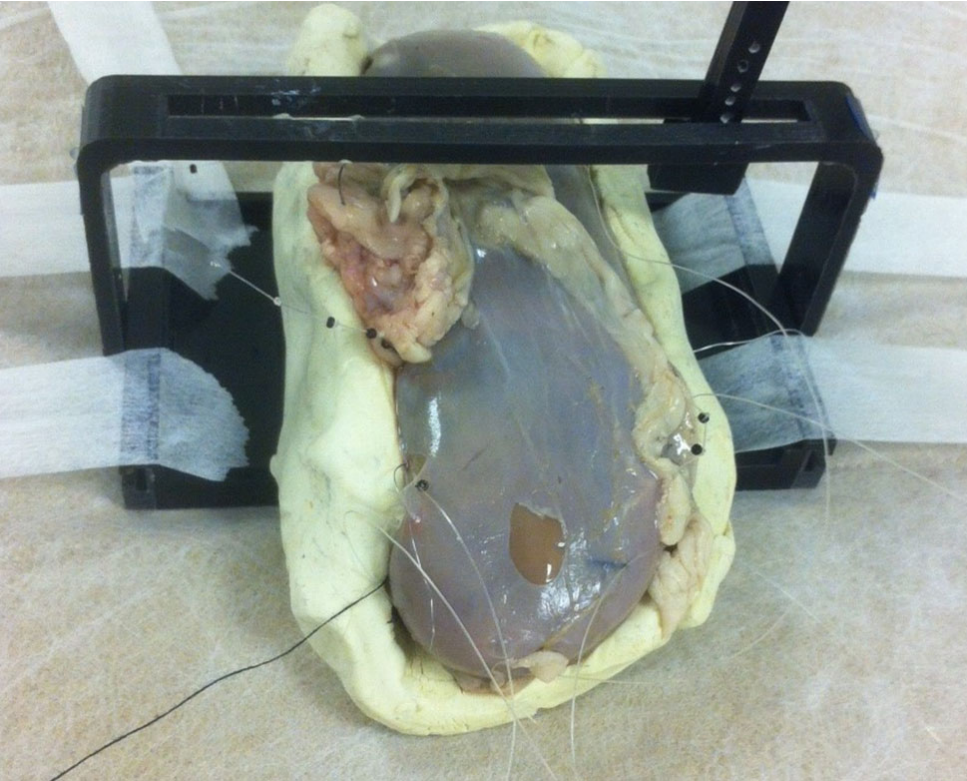
Deformation rig with kidney and support, placed on the CT scanner table

The kidney was placed on a plasticine support that acts as a complex boundary condition. An object that simulates the ultrasound transducer [14] and the user’s interaction with the platform was mounted on a 3D printed rig and used to generate the different levels of deformation. The set-up of the experimental procedure is shown in Fig. 2. The entire set-up was CT-scanned and segmented for the rest position, where the transducer was simply touching on the kidney’s surface, and under three different levels of probe-induced deformation.

### Parallel SOR solver

The simulation system is implemented within the GPU-accelerated NVIDIA FleX position-based dynamics framework [15]. For a collection of objects comprising *n* particles, the solver receives as input a vector of particle positions $\mathbf x = [\mathbf x _{1}, \mathbf x _{2}, \ldots , \mathbf x _{n}]^{\mathrm{T}}$ and a set of constraints that describe the objects to be simulated. This implementation solves a nonlinear system of equality and inequality constraints so that

1 $$\begin{aligned} C_{i}(\mathbf x +\Delta \mathbf x )= & {} 0, \quad i = 1, \ldots , n \end{aligned}$$

2 $$\begin{aligned} C_{j}(\mathbf x +\Delta \mathbf x )\ge & {} 0, \quad j = 1, \ldots , n \end{aligned}$$

Constraints are solved using the Gauss–Seidel method. For each iteration, through a linearisation of *C* around $\mathbf x$, each constraint can be solved sequentially

3 $$\begin{aligned} C_{i}(\mathbf x +\Delta \mathbf x )\approx C_{i}(\mathbf x ) + \nabla C_{i} (\mathbf x )\Delta \mathbf x =0 \end{aligned}$$

The position displacement $\Delta \mathbf x$ is restricted to lie along the constraint gradient and is weighted by the inverse of the mass matrix $M = \hbox {diag}(m_{1}, \ldots , m_{n})$,

4 $$\begin{aligned} \Delta \mathbf x =\mathbf M ^{-1} \nabla C_{i}(\mathbf x )^{\mathrm{T}} \lambda _{i} \end{aligned}$$

where $\lambda _{i}$ is a Lagrange multiplier [13] that can be computed by combining Eqs. (3) and (4)

5 $$\begin{aligned} \lambda _{i} =-\frac{C_{i} (\mathbf x )}{\nabla C_{i} (\mathbf x )\mathbf M ^{-1}\nabla C_{i} (\mathbf x )^{\mathrm{T}}} \end{aligned}$$

After each constraint has been processed, positions are updated. After a specified number of iterations, the change in velocity is determined by the total constraint displacement

6 $$\begin{aligned} \Delta \mathbf v = \frac{\Delta \mathbf x }{\Delta t} \end{aligned}$$

where $\Delta t$ is the time step. The solution $\Delta \mathbf x$ is the variation in position that attempts to satisfy the constraints. From Eq. (6), the resulting variation in position is equivalent to applying an impulse at the beginning of each time step. Using this formulation, the problem is equivalent to finding the minimum change in kinetic energy that satisfies the constraints, which is consistent with Gauss’ principle of least constraints. To accelerate this methodology, constraints are solved in a projected Gauss–Jacobi fashion. To assure convergence, constraints are averaged, i.e. each constraint is processed in parallel and position variations $\Delta \mathbf x$ are stored for each particle. At the end of each iteration, each particle’s total constraint variation is divided by the total number of constraints affecting it. To assure that this averaging is not too aggressive and the number of necessary iterations to reach a solution does not increase, a global parameter *w* is introduced to control the rate of successive over-relaxation (SOR),

7 $$\begin{aligned} \Delta \mathbf x _{i} = \frac{w}{n} \sum _{n} \nabla C_{j} \lambda _{j} \end{aligned}$$

with $w\in [1,2]$. Constraints may also be applied in groups when precedence is desired. The reader is advised to refer to the work of Müller et al. [10] and Macklin et al. [16] for further details on this approach.

Algorithm 1 represents a single simulation time step in this PBD approach. Steps (1)–(5) perform an integration step on the velocity and position estimates for each particle and take into account any external forces (e.g. gravity). Steps (6)–(9) handle contact detection. Position estimates are manipulated in steps (10)–(15) such that they satisfy the constraints. In steps (16)–(22), each constraint is processed in parallel and each particle accumulates a position estimate. After all constraints have been applied, they are averaged amongst each position. Steps (23)–(26) update velocities and positions before the end of each time step.
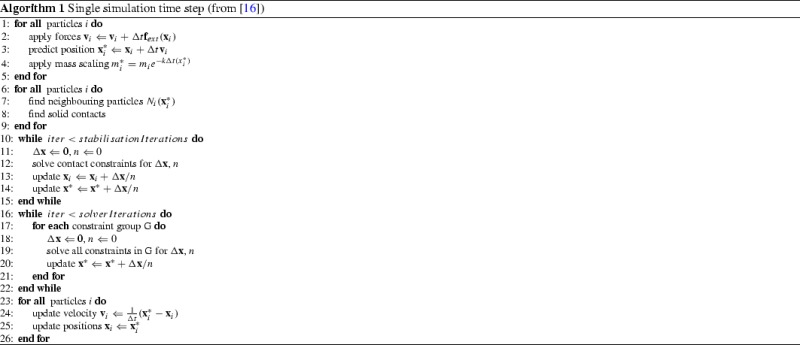


### Shape matching

In order to simulate deformable objects, the PBD approach relies on a geometrically motivated shape-matching technique. This method is based on finding the least squares optimal rigid transformations in 3D between two point sets with a priori known correspondence [17]. The algorithm requires as input a set of particles with masses $m_{i}$ and their respective initial positions $\mathbf x ^{0}_{i}$. At each time step, the original shape $\mathbf x ^{0}_{i}$ is matched to the deformed shape $\mathbf x _{i}$. Then, the deformed points $\mathbf x _{i}$ are forced towards the goal positions $\mathbf g _{i}$. Given two sets of points $\mathbf x ^{0}_{i}$ and $\mathbf x _{i}$, the minimisation problem is given by

8 $$\begin{aligned} \sum \limits _{i}m_{i}\left( \mathbf R \left( \mathbf x ^{0}_{i}- \mathbf t ^{0}\right) +\mathbf t - \mathbf x _{i}\right) ^{2} \end{aligned}$$

where $m_{i}$ are the individual masses, $\mathbf R$ is the rotation matrix, $\mathbf t$ and $\mathbf t ^{0}$ are the translation vectors. These translation vectors are defined as the centre of mass of the initial $\mathbf x _{cm}^{0}$ and actual $\mathbf x _{cm}$ shapes, respectively. Once the optimal rotation **R** and translation vector are derived, the goal positions can be computed as

9 $$\begin{aligned} \mathbf g _{i}=\mathbf R \left( \mathbf x ^{0}_{i}- \mathbf x _{cm}^{0}\right) +\mathbf x _{cm} \end{aligned}$$

From the goal positions, an integration scheme can be defined

10 $$\begin{aligned} \mathbf{v }_{i}(t+\Delta t)= & {} \mathbf{v }_{i}(t)+\alpha \frac{\mathbf{g }_{i}(t)-\mathbf{x }_{i}(t)}{\Delta t}+\Delta t \mathbf{f }_{ext}(t)/m_{i}\end{aligned}$$

11 $$\begin{aligned} \mathbf{x }_{i}(t+\Delta t)= & {} \mathbf{x }_{i}(t)+\Delta t\mathbf{v }_{i}(t+\Delta t) \end{aligned}$$

where $\alpha \in [0,1]$ simulates stiffness. This scheme is unconditionally stable and does not introduce damping, as long as the external forces are independent of the points’ locations or are applied only instantaneously [17].

### Cluster-based deformation

The implementation of the algorithm described in the previous section allows only for small deformations from the initial shape. For larger deformations, i.e. to model soft tissue, the set of particles that comprise an object can be divided into overlapping clusters. Having a surface mesh as input, the space around it is divided into overlapping cubical regions. For each region, a cluster is defined containing all the vertices that lie within it. For each time step, the original shape of each cluster is matched with the current shape. Using this formulation, each cluster assures that the following term is added to all particles that lie within it

12 $$\begin{aligned} \Delta \mathbf{v }_{i} = {\alpha \frac{\mathbf{g }_{i}^{c}(t)- \mathbf{x }_{i}(t)}{\Delta t}} \end{aligned}$$

where $\mathbf{g }_{i}^{c}(t)$ is the goal position of the particle *i* in relation to the cluster *c*.

### Collision and data preparation

The FleX engine supports several collision primitives, including static triangular meshes, dynamic convex meshes (specified as the intersection of half-spaces), signed distance fields (SDFs) and static planes. The support structure is represented as a static triangular mesh. The original 3D CT scan was first segmented in ITK-SNAP [18], exported as an STL mesh into MeshLab [19], where it was smoothed using the volume-preserving Humphrey’s Classes (HC) Laplacian smoothing algorithm, and then underwent quadric edge collapse decimation (quality threshold 0.99), producing a collision mesh with some 5000 faces [20].

**Fig. 3:**
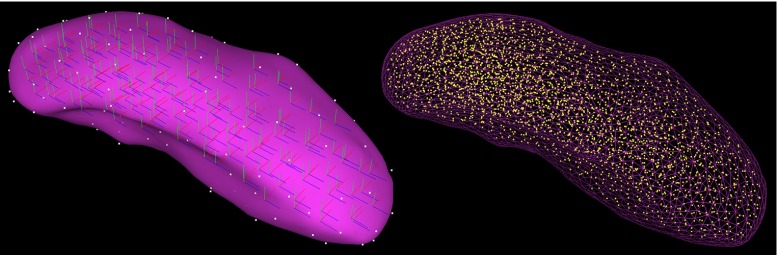
Virtual kidney model. Representation of the local coordinate systems of each cluster and tetrahedra vertices (*left*); representation of particle distribution with a radius of 2.7 mm and wireframe surface (*right*)

The geometry for the initial configuration of the kidney was prepared in a similar manner. The algorithm that computes the kidney particles generates a high-resolution voxelisation of the mesh, places temporary samples at each occupied voxel and also optionally distributes sample points randomly on the mesh surface. Particles are then introduced one at a time at sample points, and the process greedily removes any samples that fall within the bounds of the former, until no samples remain. The particles are then clustered together using the same method into shape-matching clusters. The result of this procedure is illustrated in Fig. 3. The ultrasound transducer [14] was approximated as a cuboid and realised in FleX as a dynamic convex mesh, i.e. the intersection of three parallel pairs of orthogonal half-spaces.

### Weighted matrix blending

Both the vertices of the triangular mesh representing the kidney surface and the initial fiducial positions are expressed in terms of local particle positions through weighted matrix blending, sometimes referred to as ‘skinning’ [21]. This way, the surface and subsurface geometries attached to the particle system deform in accordance with the manipulated kidney parenchyma. Each ‘skinned’ vertex can be associated with up to four local cluster coordinate frames. Weights fall off inversely with the square of the distance from vertex to local cluster origin. The same technique is used to embed a course tetrahedral mesh within the particle system (see white vertices in the left side of Fig. 3), such that a planar discretisation of the ultrasound scanning plane can be expressed barycentrically in terms of those embedded vertices, and then mapped back (i.e. undeformed) to voxels within the coordinate frame of the initial 3D CT acquisition. In this manner, ultrasound simulation scanning of a deformable kidney, or organs more generally, is realised.

**Table 1:** Simulation settings for the calibration process

Time step	1/60 s
Simulation substeps	3 (collision detection is performed once per substep)
Substep iterations	9 (each substep performs this many solve passes over the constraints)
Cluster spacing factor	3.33 (applied to particle radius, controls cluster overlap)
Volume sampling factor	4 (controls particle density)
Relaxation type	Local (relaxation $\hbox {factor} = 1.0$)
Acceleration due to gravity	$9.81\,\hbox {m/s}^2$
Tissue density	$1.05\,\hbox {g/cm}^3$ [22]
Shape friction coefficient	0.35
Particle friction coefficient	0.25
Damping factor	12.0

### Calibration

In this work, a simple one-dimensional exhaustive search was used to determine the optimal cluster stiffness coefficient ($\alpha$) for a given particle radius. The quantity undergoing minimisation was the mean fiducial error over all of the deformation stages, that is, the mean of the Euclidean distances between the segmented and simulated fiducial positions at each stage. The simulation was sufficiently fast so as not to require a more efficient search strategy. Each evaluation began with 120 time steps of simulation ‘warm-up’—that is, a conservative period during which the particles adopt their initial equilibrium positions with respect to the prevailing collision constraints. Subsequently, the ultrasound transducer position was interpolated linearly over a sequence of 90 time steps at each deformation stage, in accordance with its relative position in the corresponding 3D CT acquisition. For more complex particle behaviours with additional parameters, the calibration scheme generalises naturally to a multi-dimensional optimisation problem. Table 1 shows the FleX settings [15] used during the calibration process.

## Results

**Fig. 4:**
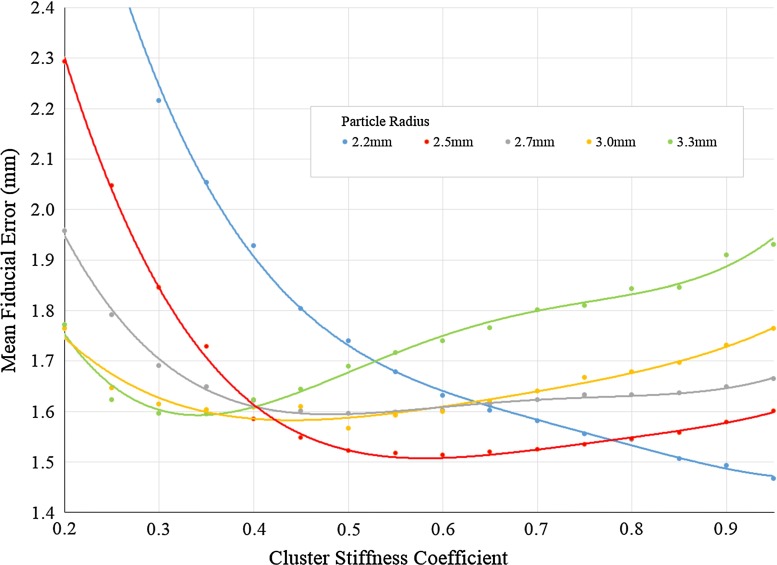
Mean fiducial error as a function of cluster stiffness, for different values of the particle radius

The graph presented in Fig. 4 represents the mean fiducial error as a function of the cluster stiffness coefficient, for different values of particle radius. The different solid lines represent the quartic polynomials fitted to smooth results.

**Table 2:** Fiducial mean error (mm)

Particle radius (mm)	2.2	2.5	2.7	3.0	3.3
Stiffness $\alpha$	0.95	0.60	0.50	0.45	0.35
0th deformation (0.00 mm)	0.62	0.65	0.72	0.79	0.93
1st deformation (5.95 mm)	1.41 (24 %)	1.49 (25 %)	1.55 (26 %)	1.51 (25 %)	1.46 (24 %)
2nd deformation (9.48 mm)	1.46 (15 %)	1.61 (17 %)	1.70 (18 %)	1.74 (18 %)	1.67 (18 %)
3rd deformation (12.38 mm)	2.26 (18 %)	2.30 (19 %)	2.41 (19 %)	2.39 (19 %)	2.32 (19 %)
Overall mean deformation	1.44	1.51	1.60	1.61	1.60

**Table 3:** Fiducial error standard deviation (mm)

Particle radius (mm)	2.2	2.5	2.7	3.0	3.3
Stiffness $\alpha$	0.95	0.60	0.50	0.45	0.35
0th deformation (0.00 mm)	0.34	0.36	0.41	0.43	0.41
1st deformation (5.95 mm)	0.59	0.65	0.64	0.61	0.58
2nd deformation (9.48 mm)	0.80	0.96	0.95	1.03	0.79
3rd deformation (12.38 mm)	1.02	1.16	1.14	1.15	1.00

**Table 4:** Fiducial maximum error (mm)

Particle radius (mm)	2.2	2.5	2.7	3.0	3.3
Stiffness $\alpha$	0.95	0.60	0.50	0.45	0.35
0th deformation (0.00 mm)	1.24	1.37	1.42	1.57	1.76
1st deformation (5.95 mm)	2.92	2.93	2.88	2.91	2.89
2nd deformation (9.48 mm)	3.16	3.97	3.80	4.21	3.33
3rd deformation (12.38 mm)	4.54	4.76	4.73	5.29	4.55

**Fig. 5:**
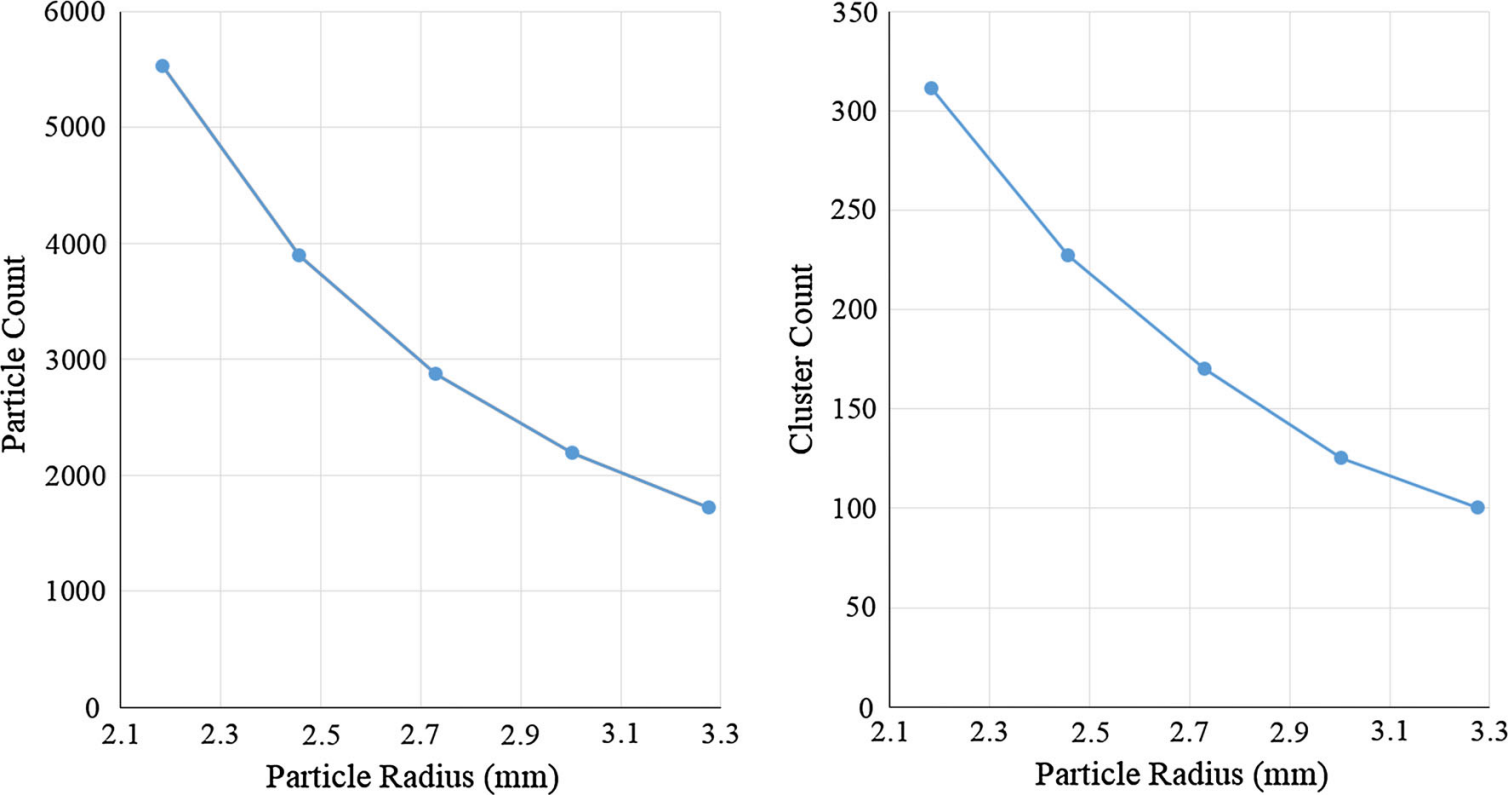
Particle count as a function of the particle radius (*Left*); cluster count as a function of the particle radius (*right*)

**Fig. 6:**
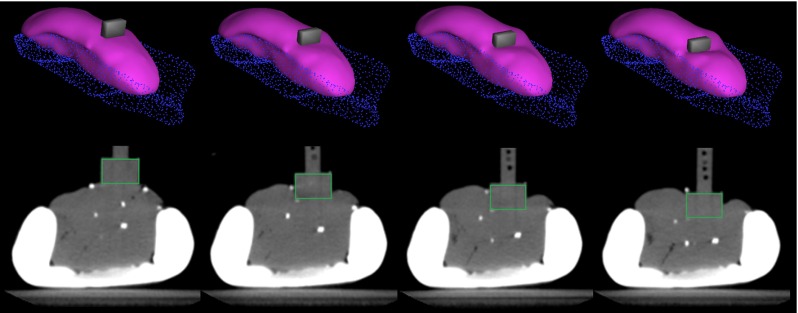
From *left* to *right*—representation of the rest position and the three increasing levels of deformation in the simulation framework (*top*) and in the CT images of the experimental set-up (*bottom*)

**Fig. 7:**
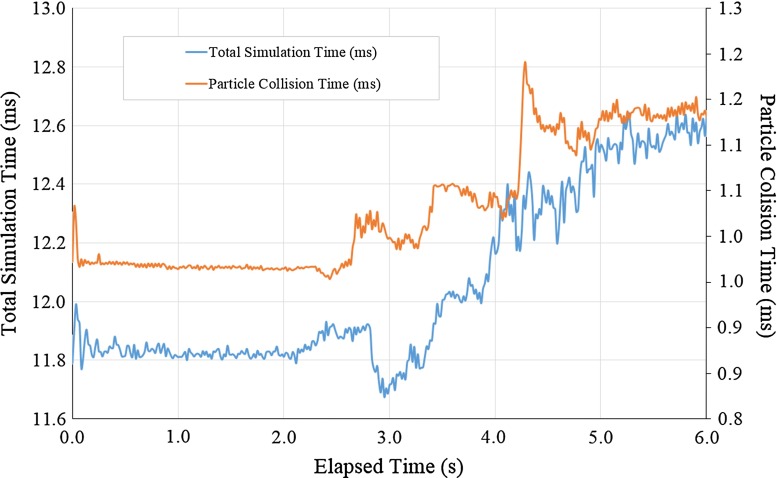
Total simulation time for one time step as a function of the simulation elapsed time, for a particle radius of 2.2 mm and cluster stiffness coefficient of 0.5

**Fig. 8:**
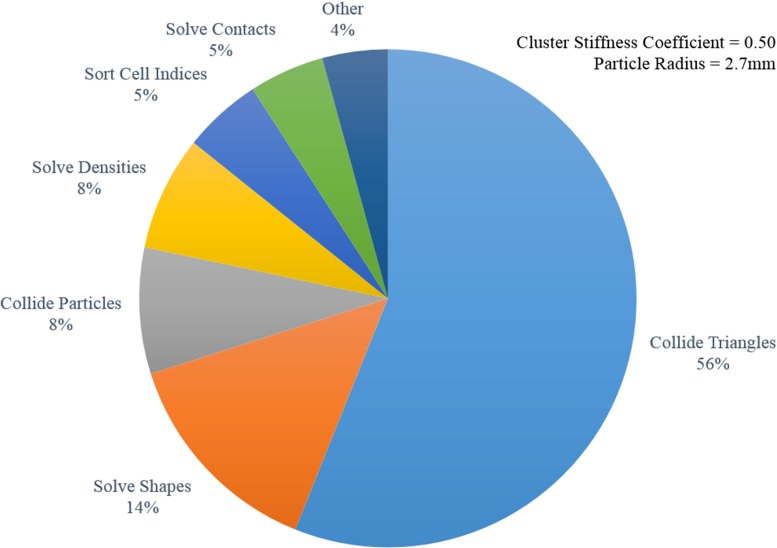
Distribution of the performance timings for the various steps of the PBD approach. A cluster stiffness coefficient of 0.5 and a particle radius of 2.7 mm were used as simulation parameters

Tables 2, 3 and 4 show the fiducial mean errors, standard deviation and maximum error, respectively, for the initial position and the different levels of deformation per particle radius. Optimal stiffness coefficients, within the permitted range [0, 1], are also tabulated. An observation of Table 2 reveals intuitive behaviour: as the deformation of the model increases, the mean fiducial error increases in kind. Moreover, this is independent of the stiffness value. The values displayed in brackets represent the percentage error with respect to the cumulative deformation and allow one to infer that the mean fiducial error is approximately proportional to the induced level of deformation. The mean fiducial error displacements are acceptable values in the context of the 12.38 mm overall displacement reached by the probe. The remaining information in this table shows that as a general trend, when the radius increases and optimal stiffness decreases, the fiducial mean error gradually increases with a range of approximately 0.15 mm. Tables 3 and 4 support the aforementioned results and illustrate a distribution of low standard deviations over all fiducials. Further analysis of the mean fiducial error as a function of the distance to the probe, across all levels of deformation, indicates that these variables are weakly correlated.

Figure 5 illustrates the number of clusters (right) and number of particles (left) as a function of the particle radius. As the particle radius increases, a decrease in the particle and cluster counts is observed. The cluster allocation algorithm results in a number of clusters proportional to the number of particles.

Figure 6 shows a representation of the deformation applied on the kidney in the CT images (bottom) and the corresponding deformation in the simulation framework (top). One can also observe the fiducial displacements as the deformation is induced.

Regarding the simulation performance, Fig. 7 shows the total simulation time as a function of the elapsed time. Two series of data are analysed—the total simulation time and the particle collision times. A ‘warm-up’ period of 2*s* is observed with no transducer displacement, during which both data series present a noisy but static mean performance time. As the transducer starts moving and induces deformation on the kidney model, an increase in the total simulation times is experienced in both data series. This is an expected result as more contacts are made.

A breakdown of the simulation performance times can be observed in Fig. 8. The total simulation time is split into the individual timings necessary in the various steps of the PBD approach. Table 5 shows the average, standard deviation, maximum and minimum performance simulation times within the simulation calibration as a function of the particle radius. As the particle radius increases, the total simulation time gradually increases. This is a counter-intuitive result, as one would expect to observe a better performance as the number of particles decreases. An inefficient underutilisation of the GPU might be an explanation for this performance behaviour. However, real-time operation is evident in general. Even in the worst-case scenario (15.68 ms), an execution rate of approximately 64 simulation steps-per-second is achieved.

**Table 5:** Time taken to simulate one time step over a single calibration evaluation (ms)

Particle radius (mm)	2.2	2.5	2.7	3.0	3.3
Mean	12.10	12.10	12.37	12.66	14.16
Standard deviation	0.33	0.33	0.21	0.29	0.29
Minimum	11.68	11.68	11.96	12.28	13.67
Maximum	12.77	12.77	12.84	13.37	15.68

## Conclusions

A novel PBD implementation [16] coupled with a clustered shape-matching constraints methodology has been shown to be capable of modelling soft tissue deformation, in the context of RAPN simulation. Coupled to this framework, the platform furthermore simulates intracorporeal 2D ultrasound image acquisition, using the preoperative CT images as the data source. Simulation stiffness and particle radius parameters were varied through the computation of multiple simulations to understand the optimal trade-off between accuracy and performance.

Results show real-time performance, accuracy and inherent unconditional stability as a result of using a shape-matching technique. The methodology developed in this framework can be applied in various other surgical simulation applications. This system requires as input a set of preoperative images and segmented structures of anatomical interest and outputs a 3D model capable of soft tissue deformation and interaction. This straightforward data preparation makes patient-specific simulation possible on a broad scale. The overall mean fiducial error was found to be compatible with the level of ultrasound probe calibration error typically observed in clinical practice [14]. The nonzero mean fiducial error measured for the 0th level of deformation might be a cause for a higher fiducial error across all deformations due to overlapping support segmentations or an absence of initial gravity compensation. These characteristics will be accounted for in future work. The exhaustive search for optimal stiffness parameter allowed an accurate and stable simulation of deformation.

Regarding the simulation performance analysis, results reveal that this implementation runs in real time while accurately maintaining complex soft tissue behaviour and boundary conditions. A PBD implementation, despite presenting the previously mentioned advantages over other approaches [16], has some limitations. The model’s realised stiffness does not depend only on the defined stiffness parameter, as it is dependent also on the time step, the number of solver iterations and the adopted number of clusters and shape-matching constraints. As a result, stiffness must be set in the context of these chosen values.

Focusing on the applicability of this simulation to patient-specific scenarios, two issues should be made clear. Previous research has been conducted in order to assess the suitability of using porcine renal tissue as a surrogate for the human kidney. Tests employing dynamic mechanical load were carried out on renal parenchyma [23] and the kidney capsule [24] to assess their behavioural response. Regarding tissue properties, the work developed by Miller et al. [25] concludes that, since kidneys and similar organs are considered almost incompressible, the dependence on the volumetric response is of minor consequence for soft organ biomechanics. Such problems are considered in light of pure-displacement or displacement-zero traction (deformation is modelled as forced motion in response to changing boundary conditions), and therefore, it is possible to model deformation without full knowledge of patient-specific tissue properties. As future work, effort will be focused on developing the planning of optimal transducer trajectories and viewpoints. Furthermore, the simulation will act as a validation context for manually operated freehand 3D acquisitions and reconstructions.

## Electronic supplementary material

Below is the link to the electronic supplementary material.

Supplementary material 1 (mp4 26545 KB)

## References

[1] Zevin B, Aggarwal R, Grantcharov TP (2014). Surgical simulation in 2013: why is it still not the standard in surgical training?. J Am Coll Surg.

[2] Isotani S, Shimoyama H, Yokota I, China T, Hisasue S, Ide H, Muto S, Yamaguchi R, Ukimura O, Horie S (2015). Feasibility and accuracy of computational robot-assisted partial nephrectomy planning by virtual partial nephrectomy analysis. Int J Urol.

[3] Makiyama K, Nagasaka M, Inuiya T, Takanami K, Ogata M, Kubota Y (2012). Development of a patient-specific simulator for laparoscopic renal surgery. Int J Urol.

[4] Figueroa-Garcia I, Peyrat J-M, Hamarneh G, Abugharbieh R (2014) Biomechanical kidney model for predicting tumor displacement in the presence of external pressure load. In: 2014 IEEE 11th international symposium on biomedical imaging (ISBI). IEEE, pp 810–813

[5] Chatelain P, Krupa A, Navab N (2015) 3D ultrasound-guided robotic steering of a flexible needle via visual servoing. In: IEEE International Conference on Robotics and Automation, ICRA’15

[6] Goksel O, Salcudean SE (2009). B-mode ultrasound image simulation in deformable 3-D medium. IEEE Trans Med Imaging.

[7] Mehrdad S, Seyed-Ahmad A, Raphael P, Nassir N, Wolfgang W (2015) Patient-specific 3D ultrasound simulation based on convolutional ray-tracing and appearance optimization. In: Medical image computing and computer-assisted intervention–MICCAI 2015. Springer, pp 510–518

[8] Nealen A, Müller M, Keiser R, Boxerman E Carlson M (2006) Physically based deformable models in computer graphics. In: Computer graphics forum, vol 25. Wiley Online Library, pp 809–836

[9] Bender J, Müller M, Otaduy MA, Teschner M, Macklin M (2014) A survey on position-based simulation methods in computer graphics. In: Computer graphics forum, vol 33. Wiley Online Library, pp 228–251

[10] Müller M, Heidelberger B, Hennix M, Ratcliff J (2007). Position based dynamics. J Vis Commun Image Represent.

[11] Kubiak B, Pietroni N, Ganovelli F, Fratarcangeli M (2007) A robust method for real-time thread simulation. In: Proceedings of the 2007 ACM symposium on Virtual reality software and technology. ACM, pp 85–88

[12] Wang Y, Xiong Y, Xu K, Tan K, Guo G (2006) A mass-spring model for surface mesh deformation based on shape matching. In: GRAPHITE, vol 6, pp 375–380

[13] Bender J, Koschier D, Charrier P, Weber D (2014). Position-based simulation of continuous materials. Comput Graph.

[14] Hughes-Hallett A, Pratt P, Mayer E, Di Marco A, Yang G-Z, Vale J, Darzi A (2014). Intraoperative ultrasound overlay in robot-assisted partial nephrectomy: first clinical experience. Eur Urol.

[15] NVIDIA Gameworks. Nvidia FleX. https://developer.nvidia.com/flex

[16] Macklin M, Müller M, Chentanez N, Kim T-Y (2014). Unified particle physics for real-time applications. ACM Trans Graph (TOG).

[17] Müller M, Heidelberger B, Teschner M, Gross M (2005) Meshless deformations based on shape matching. In: ACM Transactions on Graphics (TOG), vol 24. ACM, pp 471–478

[18] Yushkevich PA, Piven J, Hazlett HC, Smith RG, Ho S, Gee JC, Gerig G (2006). User-guided 3D active contour segmentation of anatomical structures: significantly improved efficiency and reliability. Neuroimage.

[19] Visual Computing Lab ISTI CNR. Meshlab. http://meshlab.sourceforge.net/

[20] Sastry SP, Kim J, Shontz SM, Craven BA, Lynch FC, Manning KB, Panitanarak T (2013) Patient-specific model generation and simulation for pre-operative surgical guidance for pulmonary embolism treatment. In: Image-based geometric modeling and mesh generation. Springer, pp 223–249

[21] Kavan L (2014) Part I: direct skinning methods and deformation primitives. In: ACM SIGGRAPH 2014 course—skinning: real-time shape deformation, pp 1–11

[22] Pop M, Davidson SRH, Gertner M, Jewett MlAS, Sherar MlD, Kolios MC (2010) A theoretical model for RF ablation of kidney tissue and its experimental validation. In: Biomedical simulation. Springer, pp 119–129

[23] Snedeker JG, Barbezat M, Niederer P, Schmidlin FR, Farshad M (2005). Strain energy density as a rupture criterion for the kidney: impact tests on porcine organs, finite element simulation, and a baseline comparison between human and porcine tissues. J Biomech.

[24] Snedeker JG, Niederer P, Schmidlin FR, Farshad M, Demetropoulos CK, Lee JB, Yang KH (2005). Strain-rate dependent material properties of the porcine and human kidney capsule. J Biomech.

[25] Miller K, Jia L (2013). On the prospect of patient-specific biomechanics without patient-specific properties of tissues. J Mech Behav Biomed Mater.

